# ARK: Aggregation of Reads by K-Means for Estimation of Bacterial Community Composition

**DOI:** 10.1371/journal.pone.0140644

**Published:** 2015-10-23

**Authors:** David Koslicki, Saikat Chatterjee, Damon Shahrivar, Alan W. Walker, Suzanna C. Francis, Louise J. Fraser, Mikko Vehkaperä, Yueheng Lan, Jukka Corander

**Affiliations:** 1 Dept of Mathematics, Oregon State University, Corvallis, United States of America; 2 Dept of Communication Theory, KTH Royal Institute of Technology, Stockholm, Sweden; 3 Microbiology Group, Rowett Institute of Nutrition and Health, University of Aberdeen, Aberdeen, United Kingdom; 4 MRC Tropical Epidemiology Group, London School of Hygiene and Tropical Medicine, London, United Kingdom; 5 Illumina Cambridge Ltd., Chesterford Research Park, Essex, United Kingdom; 6 Dept of Electronic and Electrical Engineering, University of Sheffield, Sheffield, United Kingdom; 7 Dept of Physics, Tsinghua University, Beijing, China; 8 Dept of Mathematics and Statistics, University of Helsinki, Helsinki, Finland; National Cancer Institute, UNITED STATES

## Abstract

**Motivation:**

Estimation of bacterial community composition from high-throughput sequenced 16S rRNA gene amplicons is a key task in microbial ecology. Since the sequence data from each sample typically consist of a large number of reads and are adversely impacted by different levels of biological and technical noise, accurate analysis of such large datasets is challenging.

**Results:**

There has been a recent surge of interest in using compressed sensing inspired and convex-optimization based methods to solve the estimation problem for bacterial community composition. These methods typically rely on summarizing the sequence data by frequencies of low-order *k*-mers and matching this information statistically with a taxonomically structured database. Here we show that the accuracy of the resulting community composition estimates can be substantially improved by aggregating the reads from a sample with an unsupervised machine learning approach prior to the estimation phase. The *aggregation of reads* is a *pre-processing* approach where we use a standard K-means clustering algorithm that partitions a large set of reads into subsets with reasonable computational cost to provide several vectors of first order statistics instead of only single statistical summarization in terms of *k*-mer frequencies. The output of the clustering is then processed further to obtain the final estimate for each sample. The resulting method is called Aggregation of Reads by K-means (ARK), and it is based on a statistical argument via mixture density formulation. ARK is found to improve the fidelity and robustness of several recently introduced methods, with only a modest increase in computational complexity.

**Availability:**

An open source, platform-independent implementation of the method in the Julia programming language is freely available at https://github.com/dkoslicki/ARK. A Matlab implementation is available at http://www.ee.kth.se/ctsoftware.

## Introduction

The advent of high-throughput sequencing technologies has enabled detection of bacterial community composition at an unprecedented level of detail. A technological approach is to produce for each sample a large number of reads from amplicons of the 16S rRNA gene, which enables an identification and comparison of the relative frequencies of different taxonomic units present across samples. The rapidly increasing number of reads produced per sample results in the need for fast taxonomic classification of samples. This problem has attracted considerable recent attention [1–5].

Many existing approaches to the bacterial community composition estimation problem use 16S rRNA gene amplicon sequencing where a large amount of moderate length reads (around 250–500 bp) are produced from each sample and then generally either clustered or classified to obtain a composition estimate of taxonomic units. In the clustering approach, reads are grouped into taxonomic units by either distance-based or probabilistic methods [6–8], such that the actual taxonomic labels are assigned to the clusters afterwards by matching their consensus sequences to a reference database. In contrast to the clustering methods, the classification approach is based on using a reference database directly to assign reads to meaningful biological units. Methods for the classification of reads have been based either on homology using sequence similarity, or on genomic signatures in terms of *k*-mer composition. Examples of homology-based methods include MEGAN [9, 10] and phylogenetic analysis [11]. Another popular approach is to use a Bayesian classifier [1, 12, 13]. One such method, the Ribosomal Database Project’s (RDP) naïve Bayesian classifier (NBC) [1], assigns a label explicitly to each read produced for a particular sample. Despite the methodological simplicity of NBC, the RDP classifier may still require several days to process a large data set in a desktop environment due to the read-by-read classification approach. Given this challenge, considerably faster estimation methods based on mixtures of *k*-mer counts have been developed, for example, Taxy [2], Quikr [3] and the recently proposed SEK [14]. Taxy is a convex-optimization based method. SEK and Quikr are sparse signal processing based methods (inspired by compressed sensing and convex-optimization), and SEK was shown to perform better than Quikr and Taxy in [14].

Taxy, Quikr and SEK all use as their main input a (statistical) mean vector of sample *k*-mer counts computed from the reads obtained for a sample. The *k*-mer counts (also called *k*-mers) are feature vectors extracted from raw sequence data. The necessary modeling assumption is that the sample mean vector of *k*-mer counts (that means first order statistics) is sufficiently informative about the sample composition. These three methods do not use the reads in any additional way once the mean vector of *k*-mers is computed. We propose here an alternative basis of information aggregation that remains computationally tractable to allow processing of large sets of reads. Borrowing ideas from source coding in signal processing [15, 16], clustering in machine learning and source coding [17], fusion in signal estimation [18] and divide-and-conquer based shotgun sequence assembly [19], our novel approach first segregates the full set of reads into subsets (in the *k*-mers feature space), computes the mean vector for each subset, employs a standard method (such as Taxy, Quikr or SEK) to estimate composition for each subset, and finally fuses these estimates into a composition estimate jointly for all the reads. To segregate the reads into subsets, we choose to employ the K-means clustering algorithm [20]. Since the K-means clustering algorithm is simple and computationally inexpensive for a reasonable number *Q* of clusters (subsets), it can be used to partition even fairly large sets of reads into more (intra) homogeneous subsets. By its very algorithmic nature, K-means clustering partitions the feature space into *Q* non-overlapping regions and provides a set of corresponding mean vectors. This is called *codebook generation* in vector quantization [15], originally from signal processing, coding and clustering. Our new method is termed as Aggregation of Reads by K-means (ARK). From the statistical perspective, theoretical justification of ARK stems from a modeling framework with a mixture of densities.

## Methods

### Summarizing read sequence data by single mean *k*-mer counts

In the method description, we denote the non-negative real line by ℝ_+_ and statistical expectation operator by 𝔼[.]. First, we describe the previously published approach of using single k-mer summaries for each sample. Let $\text{x}\in {\mathbb{R}}_{+}^{4^{k}}$ and 𝒞_*m*_ denote random *k*-mer feature vectors and *m*th taxonomic unit, respectively. Given a test set of *k*-mers (computed from reads), the distribution of the test set is modeled as

$$\begin{array}{c} p\left(x\right)=\sum_{m=1}^{M}p\left(C_{m}\right)\;\;p\left(x|C_{m}\right), \end{array}$$ (1)

where we denote probability for taxonomic unit *m* (or class weight) by *p*(𝒞_*m*_), satisfying $\sum_{m=1}^{M}p({𝒞}_{m})=1$. Note that ${\left\{p({𝒞}_{m})\right\}}_{m=1}^{M}$ is the composition of taxonomic units in the given test set (reads). The inference task is to estimate *p*(𝒞_*m*_) as accurately as possible with a reasonable computational resource. Let us derive the mean vector

$$\begin{array}{c} E\left[x\right]=\int x\;p\left(x\right)\;dx=\int x\sum_{m=1}^{M}p\left(C_{m}\right)\;\;p\left(x|C_{m}\right)\;dx=\sum_{m=1}^{M}p\left(C_{m}\right)\;\;\int x\;\;p\left(x|C_{m}\right)\;dx. \end{array}$$ (2)

The mean 𝔼[**x**] contains information about *p*(𝒞_*m*_) in this probabilistic formulation. In practice, the information summary is obtained by computing the sample mean from the complete set of reads available for a sample. Let us denote the sample mean of *k*-mers feature vectors of reads by $\mu \in {\mathbb{R}}_{+}^{4^{k}}$ with the assumption that ***μ*** ≈ 𝔼[**x**]. Several methods, such as Taxy [2], Quikr [3], and SEK [14] use the sample mean ***μ*** directly as the main input to compute the composition *p*(𝒞_*m*_).

### Aggregation of reads by K-means (ARK)

For the above-described principle of information aggregation from the reads by the mean vector of *k*-mer counts, computation of the sample mean vector is straightforward. This consequently enables handling of a very large amount of reads with low computational cost. However, we hypothesize that the sample mean vector computed from the full set of reads is not sufficient in terms of information content to facilitate accurate estimation of *p*(𝒞_*m*_). Indeed, since typically the number of training taxonomic units *M* is much larger than the number of *k*-mers (for example *k* = 6), the set of *k*-mer vectors for ${\left\{{𝒞}_{m}\right\}}_{m=1}^{M}$ is not linearly independent, and so we risk reconstructing a mixture of taxonomic units as a single taxonomic unit. Hence, we segregate the reads into several subsets and compute a sample mean vector separately for each subset, assuming that a set of sample mean vectors is more informative than a single mean vector. Note that in the case where the resulting read subsets were not in practice distinct from each other in terms of their *k*-mer counts, the subsequent composition estimate would effectively be identical to the estimate obtained with a single data summary described in Eqs (1) and (2).

Let us partition the *k*-mers feature space ${\mathbb{R}}_{+}^{4^{k}}$ into *Q* non-overlapping regions 𝓡_*q*_ such that $\cup_{q=1}^{Q}{𝓡}_{q}={\mathbb{R}}_{+}^{4^{k}}$ and ∀*q*, *r*, *q* ≠ *r*, 𝓡_*q*_ ∩ 𝓡_*r*_ = ∅. Such partitions can be formed by a standard K-means algorithm that typically uses a nearest neighbor classification rule based on square Euclidean distance measure. The non-overlapping regions 𝓡_*q*_ are called Voronoi regions. We define *P*_*q*_ ≜ Pr(**x** ∈ 𝓡_*q*_) satisfying $\sum_{q=1}^{Q}P_{q}=1$. In practice, *P*_*q*_ is computed as

$$\begin{array}{c} P_{q}=\frac{\text{number}\;\;\text{of}\;\;\text{feature}\;\;\text{vectors}\;\;\text{in}\;\;R_{q}}{\text{total}\;\;\text{number}\;\;\text{of}\;\;\text{feature}\;\;\text{vectors}}. \end{array}$$ (3)

It is reminded that the feature vectors are *k*-mers. The distribution of the full test set and subsets can be written as

$$\begin{array}{ccc} & & p\left(x\right)=\sum_{q=1}^{Q}P_{q}\;\;p\left(x|x\in R_{q}\right),\\ & & p\left(x|x\in R_{q}\right)=\sum_{m=1}^{M}p\left(C_{m}|x\in R_{q}\right)\;\;p\left(x|C_{m},x\in R_{q}\right), \end{array}$$ (4)

where the first equation follows a standard mixture density framework. Now, if we can estimate *p*(𝒞_*m*_∣**x** ∈ 𝓡_*q*_), then the final quantity of interest *p*(𝒞_*m*_) can be estimated as

$$\begin{array}{c} p\left(C_{m}\right)=\sum_{q=1}^{Q}P_{q}\;\;p\left(C_{m}|x\in R_{q}\right). \end{array}$$ (5)

The estimation of *p*(𝒞_*m*_) in Eq (5) is a judicious fusion of *p*(𝒞_*m*_∣**x** ∈ 𝓡_*q*_) through a linear combination. Let us now derive the mean vector for 𝓡_*q*_, which is a conditional mean vector

$$\begin{array}{ccc} & & E[x|x\in R_{q}]\\ & & =\int x\;\;p(x|x\in R_{q})\;dx\\ & & =\sum_{m=1}^{M}p\left(C_{m}|x\in R_{q}\right)\;\;\int \;x\;\;p\left(x|C_{m},x\in R_{q}\right)\;\;dx. \end{array}$$ (6)

The mean 𝔼[**x**∣**x** ∈ 𝓡_*q*_] contains information about *p*(𝒞_*m*_∣**x** ∈ 𝓡_*q*_). In practice we use the sample mean denoted by ***μ***_*q*_ with the assumption that ***μ***_*q*_ ≈ 𝔼[**x**∣**x** ∈ 𝓡_*q*_]. Comparing Eqs (2) and (6), for the *q*th Voronoi region 𝓡_*q*_ we can estimate composition *p*(𝒞_*m*_∣**x** ∈ 𝓡_*q*_) by using an appropriate composition estimation method, such as Taxy, Quikr or SEK.

### Algorithms

The ARK algorithm can be implemented by following steps.

1. Divide the full test dataset of *k*-mers into *Q* subsets. The region 𝓡_*q*_ corresponds to the *q*th subset.
2. For the *q*th subset, compute *P*_*q*_ and the sample mean ***μ***_*q*_.
3. For the *q*th subset, apply a composition estimation method that uses the input ***μ***_*q*_; estimate *p*(𝒞_*m*_∣**x** ∈ 𝓡_*q*_).
4. Estimate *p*(𝒞_*m*_) by $p({𝒞}_{m})=\sum_{q=1}^{Q}P_{q}\;\;p({𝒞}_{m}|\text{x}\in {𝓡}_{q})$.

**Fig 1:**
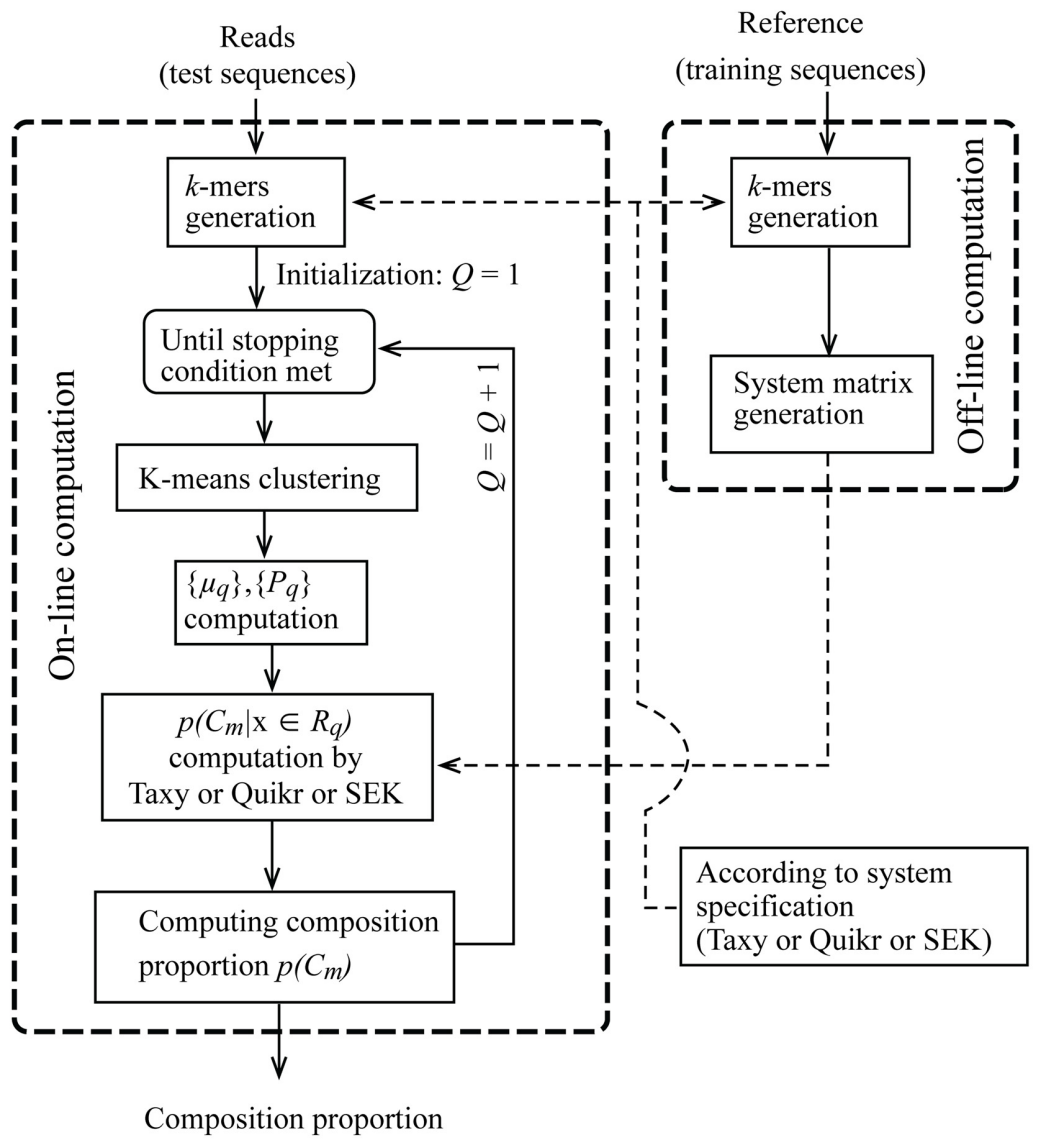
A flow-chart of the ARK method.

The ARK method is described using a flow-chart in Fig 1. The flow-chart shows the main components of the overall system and the associated off-line and on-line computations. The crucial computational/statistical challenges related to the ARK algorithm outlined above are as follows:

1. What is an appropriate number of subsets *Q*?
2. How should one form the subsets 𝓡_*q*_?

The above points are inherent to any subset forming algorithm, and more generally to any clustering algorithm. Furthermore, finding optimal regions (or clusters) requires alternative optimization techniques. Given a pre-defined *Q*, typically a K-means algorithm performs two alternating optimization steps. These are: (1) given a set of representation vectors ${\left\{\mu_{q}\right\}}_{q=1}^{Q}$ (also called code vectors) form new clusters ${\left\{{𝓡}_{q}\right\}}_{q=1}^{Q}$ by a nearest neighbor rule (or form new subsets from the full dataset), (2) find the set of cluster representation vectors given the assignment of data into clusters. The optimal representation vector is the mean vector if squared Euclidean distance is used for the nearest neighbor rule. The K-means algorithm initializes with a set of representative vectors and runs alternating optimization until convergence in the sense that the average squared Euclidean distance is no longer reduced. In the present paper we perform the clustering using a popular vector quantization method called the Linde-Buzo-Gray (LBG) algorithm [15] (or source coding literature). There are several variants of the LBG available. In one variant, the algorithm starts with *Q* = 1 and then slowly splits the dense and high probability clusters to end up with a high *Q*, such that it does not deviate significantly from an exponentially decaying bit rate versus coding distortion (rate-distortion) curve.

In ARK, we use the following two strategies to solve the two challenges listed above.

1. Optimal/deterministic strategy: Start with *Q* = 1, which corresponds to the previous approach with a single mean vector as the data summary. Then set *Q* = 2 for LBG algorithm that uses square Euclidean distance as the distortion measure; the LBG algorithm minimizes mean of square Euclidean distance (also called mean square error). Initialization is done by a standard split approach where the mean vector is perturbed. Using *Q* = 2, ${\left\{{𝓡}_{q}\right\}}_{q=1}^{2}$ is formed and we estimate *p*(𝒞_*m*_). Subsequently, *Q* is increased by one until a convergence criterion is met. For *Q* ≥ 3, we always split the highest ranking cluster into two subclusters and use the LBG algorithm to find the optimal clusters. The number of clusters *Q* is no longer increased if the estimated values of *p*(𝒞_*m*_) differ negligibly for *Q* and (*Q* − 1). In practice, the stopping condition we use is that the variational distance between *p*(𝒞_*m*_)∣_*Q*_ and *p*(𝒞_*m*_)∣_(*Q*−1)_ is less than a predetermined threshold. This condition can be written as $\sum_{m=1}^{M}\text{abs}\left(p({𝒞}_{m}){|}_{Q}-p({𝒞}_{m}){|}_{\left(Q-1\right)}\right)<\eta$, with a user defined choice of the threshold *η*. Note that *η* ∈ (0,1] provides an allowable limit as a scaled variational distance (VD) between two probability mass functions; a typical choice of *η* can be 0.01. This strategy is typically found to provide consistent performance improvement in the sense of estimating *p*(𝒞_*m*_) with the increase in *Q* by the step of one, but without absolute guarantee as the target optimization strategy minimizes mean square error. Furthermore, we allow an increment in the number of clusters up to a pre-defined maximum limit *Q*_*max*_. Typically *Q*_*max*_ is preferably chosen as an integer power of two. A typical choice of *Q*_*max*_ can be between 16 to 256.
2. Non-optimal/random strategy: For very large test sets, we use a pre-determined *Q* and a random choice of the *Q* representation vectors. Then the full test set is divided into *Q* subsets by a nearest neighbor rule and we compute the set of *Q* mean vectors {***μ***_*q*_}, and cluster probabilities {*P*_*q*_}. Even though this non-optimal strategy does not use an alternating optimization (such as LBG algorithm) to form optimal clusters, it divides the full test set into sub-sets, resulting in a set of *Q* localized mean vectors across the full test set.

Finally we mention that the use of K-means is fully motivated by its simplicity and computational ease. Use of statistical K-means in the form of expectation-maximization based mixture modeling (for example, Gaussian mixture model) could have been investigated, but requires more computation to handle a large dataset of reads.

### Synthetic data generation for method evaluation

To evaluate the performance of the ARK method, we conducted experiments for simulated data as described below. For these, and all computations reported in the remainder of the paper, we used Matlab version R2013b (with some instances of C code), on a desktop workstation with an Intel Core i7 4930K processor and 64Gb of RAM.

#### Test datasets (Reads)

We simulated 180 16S rRNA gene 454-like datasets using the RDP training set 7 and the Grinder read simulator [21] targeting the V1–V2 and V3–V5 variable regions with read lengths fixed at 250 bp or normally distributed with a mean of 450 bp and variance 50 bp. Read depths were chosen to be either 10K, 100K or 250K, while three different read distributions were used: power law, uniform, and linear. Diversity was set at either 50, 100, or 500 taxa and chimera percentages were set to 5% or 35%. The Balzer model [22] was chosen for homopolymer errors, and copy bias was included while length bias was excluded.

#### Training dataset (Reference)

In our ARK experiments we used Quikr [3] and SEK [14] to estimate *p*(𝒞_*m*_∣**x** ∈ 𝓡_*q*_). The RDP training set 7 was used as the base reference database for both Quikr and SEK. Note that this is the same as database *D*_small_ utilized in [3]. While in the main manuscript we use the same data for both training and testing the base methods (Quikr and SEK), in S1 File we include results obtained when the test datasets have taxa absent from the training database (that is, sister taxa have been excluded from the training database). As expected, all methods experience a loss in reconstruction accuracy when sister taxa are absent, but ARK Quikr and ARK SEK are still more accurate than RDP’s NBC.

### Real biological data

To further evaluate ARK, we also utilized 28 Illumina MiSeq 16S rRNA gene human body-site associated samples, plus one negative control sample. The real data consist of a total of over 5.7 M reads distributed over three variable regions (V1–V2, V3–V4, and V3–V5) as well as two body sites (vagina and feces).

For each of these samples DNA was extracted using the FastDNA SPIN Kit for Soil with a FastPrep machine (MP Biomedicals) following the manufacturer’s protocol. 16S rRNA gene amplicons were generated from the DNA extractions using the primer combinations listed in Section 5 of S1 File. The Q5 High-fidelity polymerase kit (New England Biolabs) was used to amplify the 16S rRNA genes, and PCR conditions were as follows: 98°C for 2 minutes, followed by 20 cycles of 98°C for 30 seconds, 50°C for 30 seconds and 72°C for 1 minute 30 seconds, followed by a final extension step at 72°C for 5 minutes. Following PCR, the amplicons were then purified using the Wizard SV Gel and PCR Clean-Up kit (Promega, UK). Sequencing of 16S rRNA gene amplicons was carried out by Illumina Inc. (Little Chesterford, UK) using a MiSeq instrument run for 2 x 250 (V1–V2), 300 + 200 (V3–V4) and 400 + 200 (V3–V5) cycles. These data have been submitted to the European Nucleotide Archive using the accession number PRJEB9828.

After trimming 20 bp of primer off each read, the sequences were trimmed from the right until all bases had a quality score greater than 27. This reduced the total number of reads to approximately 4M, and reduced the mean read length from 315 bp to 257 bp. We then utilized all resulting unpaired reads (both forward and reverse) including any duplicate sequences. We include in S1 File results for an alternative error-correction protocol, as well as results for assembling paired-end reads (Figs E and F in S1 File).

### Ethics Statement

For human body-site associated samples, the faecal samples used were not part of a clinical study so there is no corresponding ethical approval or written consent. There were no clinical records. The samples are anonymised and de-identified. Further, vaginal samples were collected as part of an observational microbicide feasibility study. The study was approved by the Ethics Committees of the National Institute for Medical Research in Tanzania and London School of Hygiene and Tropical Medicine, and all participants gave written informed consent. All records were anonymized and de-identified prior to this retrospective analysis.

## Results

### Performance measure and relevant methods

As a quantitative performance measure, we use variational distance (VD) to compare between known proportions of taxonomic units **p** = [*p*(𝒞_1_), *p*(𝒞_2_), …, *p*(𝒞_*M*_)]^*t*^ and the estimated proportions $\hat{\text{p}}={\left[\hat{p}({𝒞}_{1}),\;\hat{p}({𝒞}_{2}),\ldots ,\hat{p}({𝒞}_{M})\right]}^{t}$. The VD is defined as

$$\begin{array}{c} \text{VD}=0.5\times ∥p-\hat{p}{∥}_{1}\in \left[0,1\right]. \end{array}$$

A low VD indicates more satisfactory performance.

For ARK, we used both SEK and Quikr as the underlying estimation methods applied to each cluster. These recent methods were chosen as appropriate representatives of fast and accurate sparse signal processing approaches. A *k*-mer size of *k* = 6 was used for both Quikr and SEK.

As part of the SEK pipeline, sequences in a given database are split into subsequences. We selected from the 10,046 sequences in the RDP training set 7 all sequences longer than 700 bp in length, and then split the sequences into subsequences of length 400 bp with 100 bp of overlap. This corresponds to setting *L*_*w*_ = 400 and *L*_*p*_ = 100 as specified in [14]. We used the SEK algorithm ${\text{OMP}}_{\text{sek}}^{+,1}$ with parameters as in [14].

### Results for Simulated Data

#### Effect of increasing number of clusters

**Fig 2:**
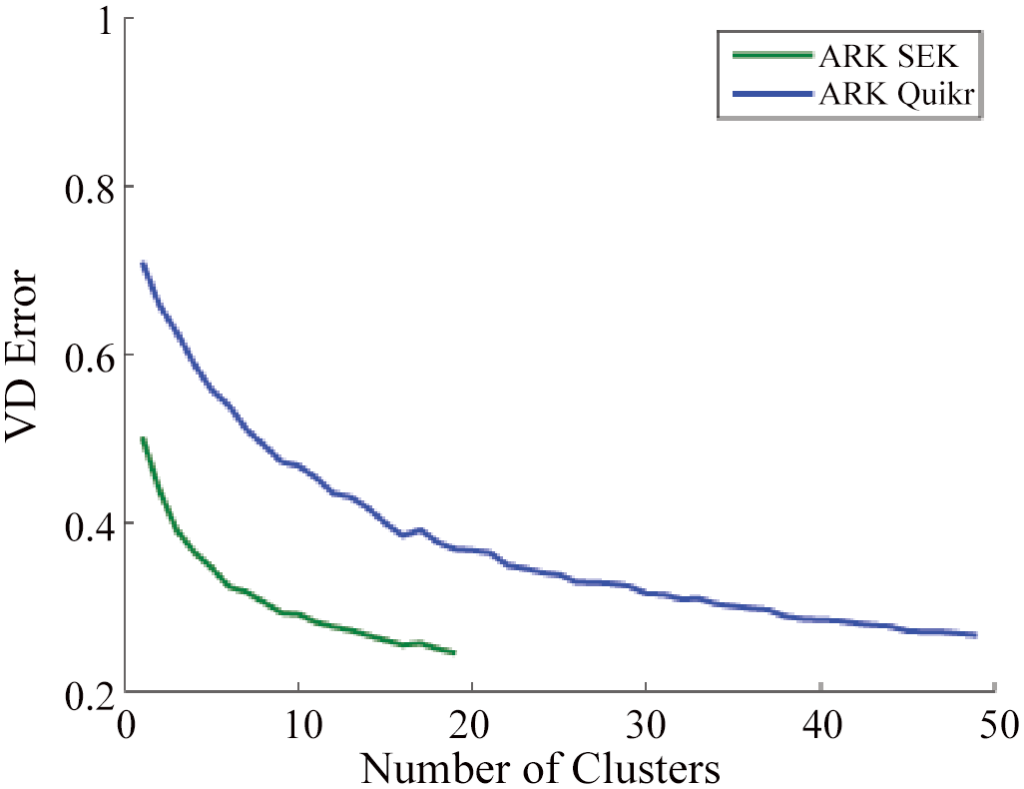
Results for the random K-means clustering on the simulated data. Mean VD error at the genus level as a function of the number of clusters. Note the improvement that ARK contributes to each method.

We first investigate how an increase in the number of clusters *Q* affects the composition reconstruction fidelity and algorithm execution time for the simulated data. Only the non-optimal/random strategy of K-means clustering was utilized as we found that the performance improvement for optimal/deterministic strategy was insignificant given the resulting increase in execution time (results not shown). Averaging the VD error at the genus level over all 180 simulated experiments, it was found that combining ARK with both SEK and Quikr resulted in a power law kind of decay of VD error as a function of the number of clusters (Fig 2). ARK causes a substantial increase in reconstruction fidelity which can be seen since using ARK SEK or ARK Quikr with one cluster is equivalent to running SEK or Quikr with no modification.

**Fig 3:**
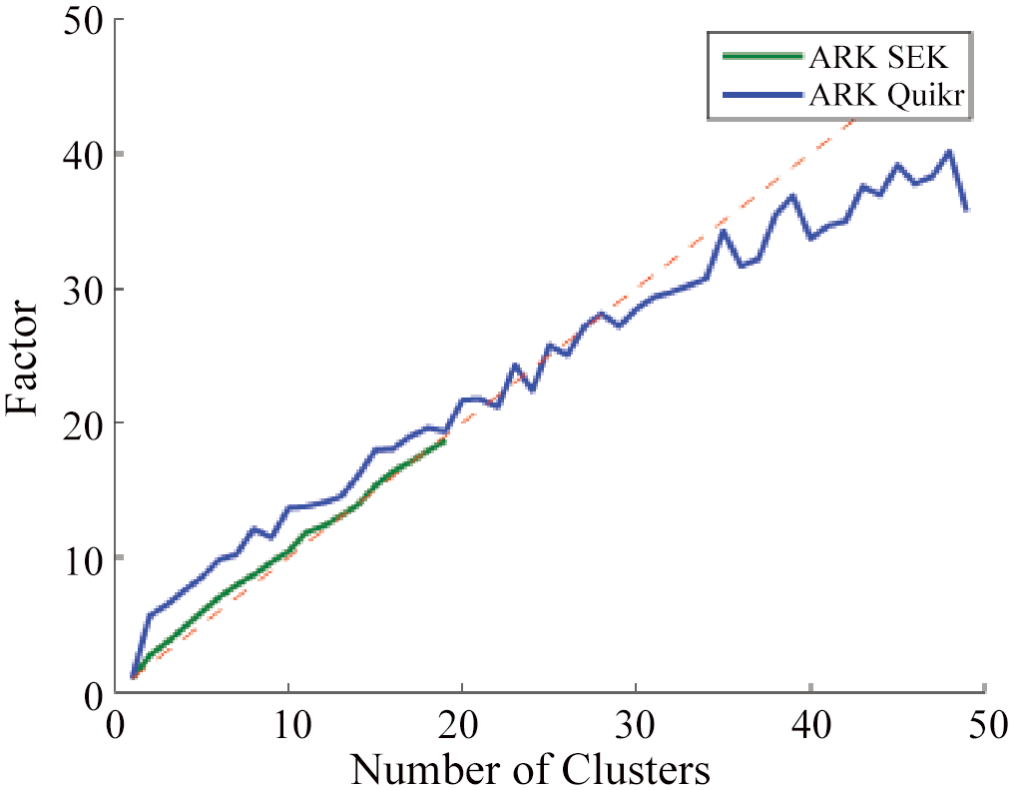
Results for the random K-means clustering on the simulated data. Mean execution time increase (factor given in comparison to running SEK or Quikr in the absence of ARK) as a function of number of clusters. The dashed line represents a line with slope 1.

Since the underlying algorithm (SEK or Quikr) must be executed on each cluster formed by the K-means clustering, we expect the total algorithm execution time to increase by a factor equal to the number of chosen clusters. As seen in Fig 3, both algorithms experience an increase in execution time roughly proportional to the number of clusters.

#### Fixed number of clusters

**Fig 4:**
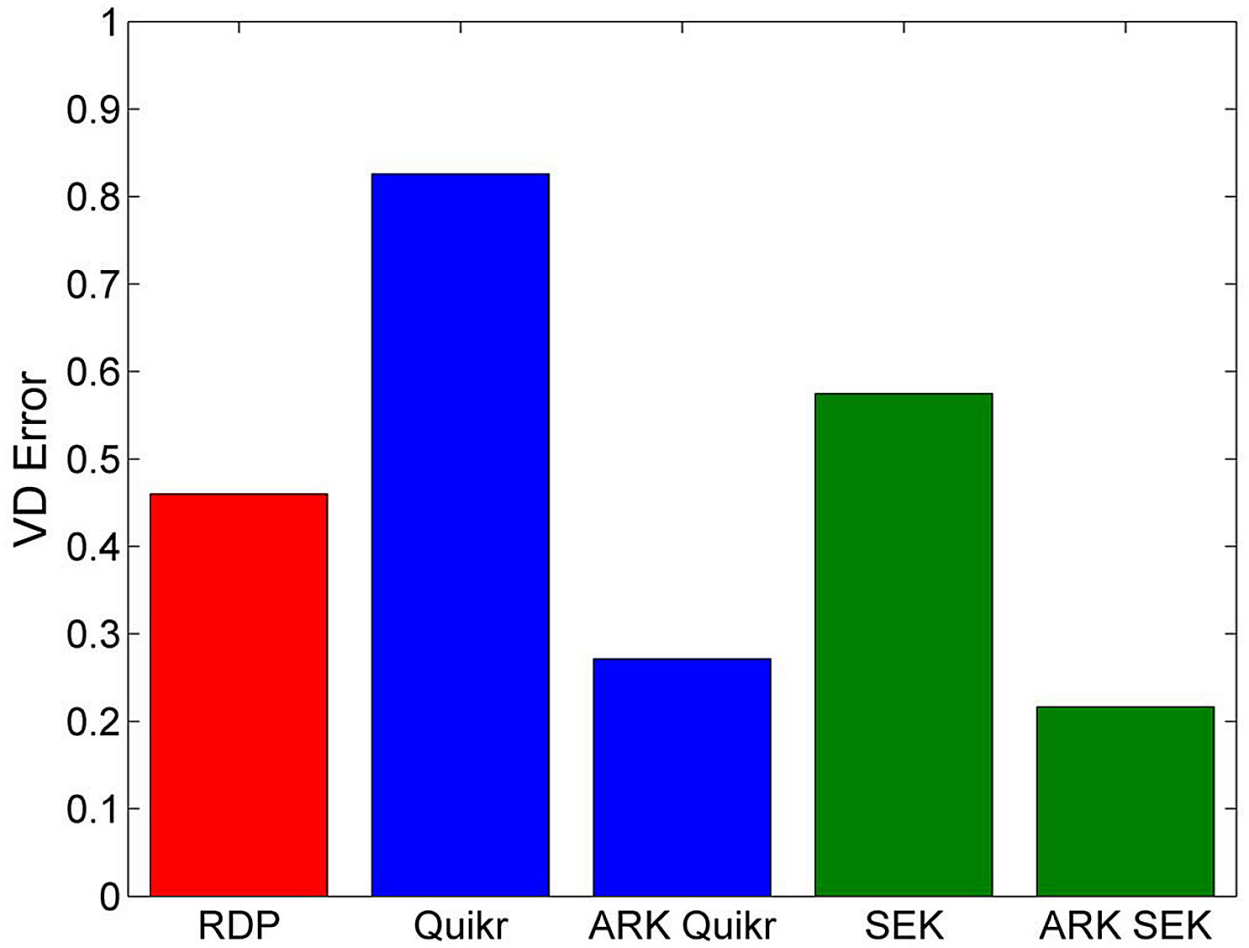
Comparison of the underlying algorithms with and without ARK. Results are for the random K-means clustering on the simulated data when fixing the number of clusters to 75. Mean VD error at the genus level. Included for comparison are results for RDP’s NBC (compare to Fig 2(b) of [3]).

**Fig 5:**
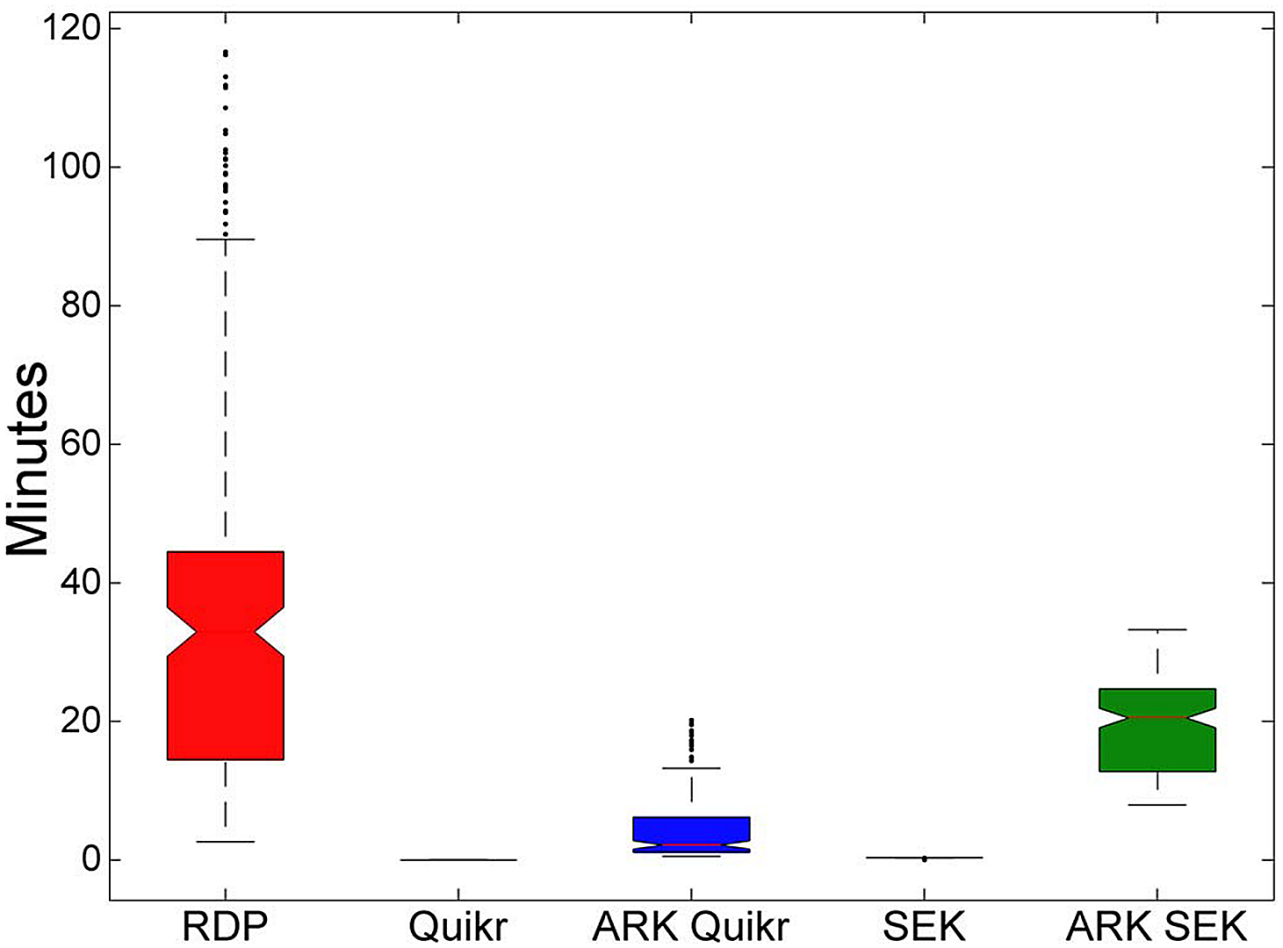
Comparison of the underlying algorithms with and without ARK. Results are for the random K-means clustering on the simulated data when fixing the number of clusters to 75. Boxplot of the individual simulated sample execution times. Mean execution times for Quikr and ARK Quikr were 1.75 seconds and 4.71 minutes, while for SEK and ARK SEK they were 21.26 seconds and 19.21 minutes respectively. Mean execution time for RDP’s NBC was 38.19 minutes.

As seen above, given the decrease in VD as a function of the number of clusters, we also fixed the number of clusters *Q* to 75 to compare the performance of the underlying algorithms with and without ARK. There was a significant decrease in the VD error (as seen in Fig 4) at the cost of an increase in execution time (as seen in Fig 5). However, given the speed of both Quikr and SEK, we expect the addition of ARK will not result in prohibitively long execution times. Indeed, as seen above, on real biological data both ARK Quikr and ARK SEK are still several hours faster than the Ribosomal Database Project’s Naïve Bayesian Classifier (RDP’s NBC) [1], even when using 75 clusters.

### Real Biological Data

**Fig 6:**
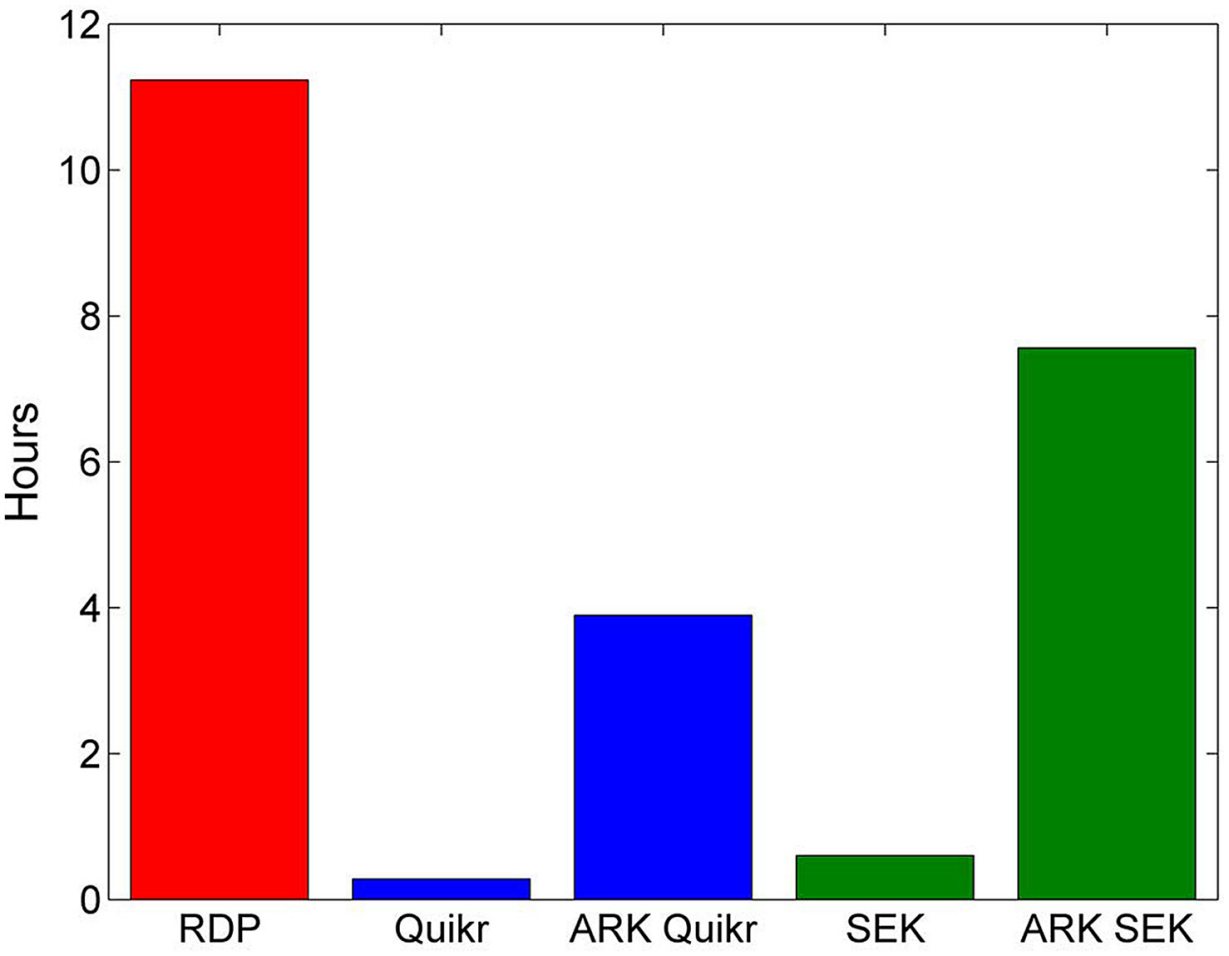
Total execution time for each method on the 28 samples of real biological data.

We used ARK combined with SEK and Quikr to analyze the real biological data and compared these results to those obtained from the RDP’s NBC. All methods used RDP’s training set 7 as the underlying training database. The random K-means clustering was used for the ARK method, and the number of clusters *Q* was set to 75. Fig 6 demonstrates the total execution time of each method. While ARK does increase the execution time of Quikr and SEK, the total execution time is still significantly less than that of RDP’s NBC. Note that all datasets here are not de-duplicated. Execution time of RDP’s NBC can be accelerated by de-duplicating the data before classifying. However, this requires additional computational time to find duplicate sequences, and since we are directly comparing classification methods here (not computational shortcuts) we use the same non-de-duplicated data for all methods.

**Fig 7:**
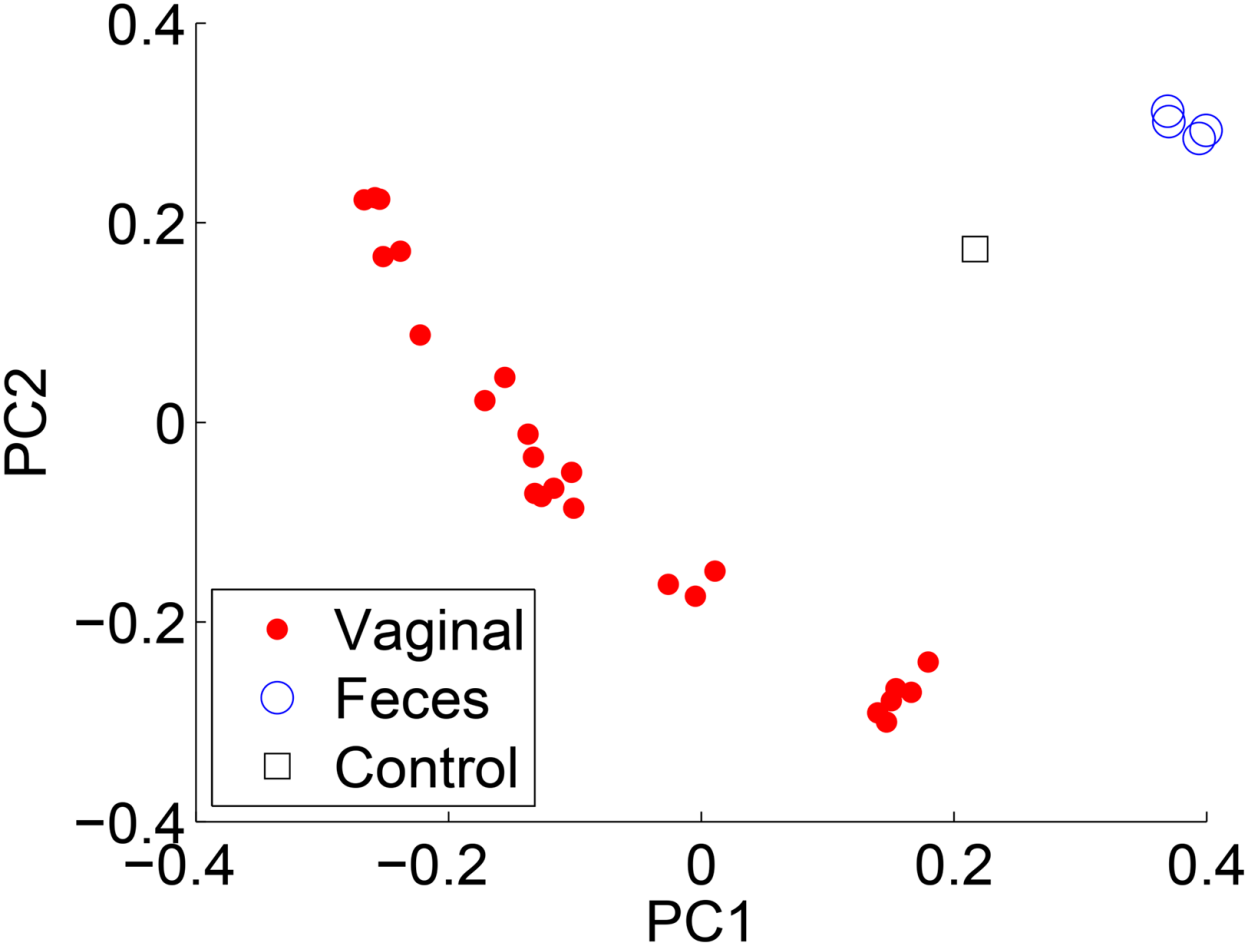
PCoA plots using the Jensen-Shannon divergence for RDP’s NBC.

**Fig 8:**
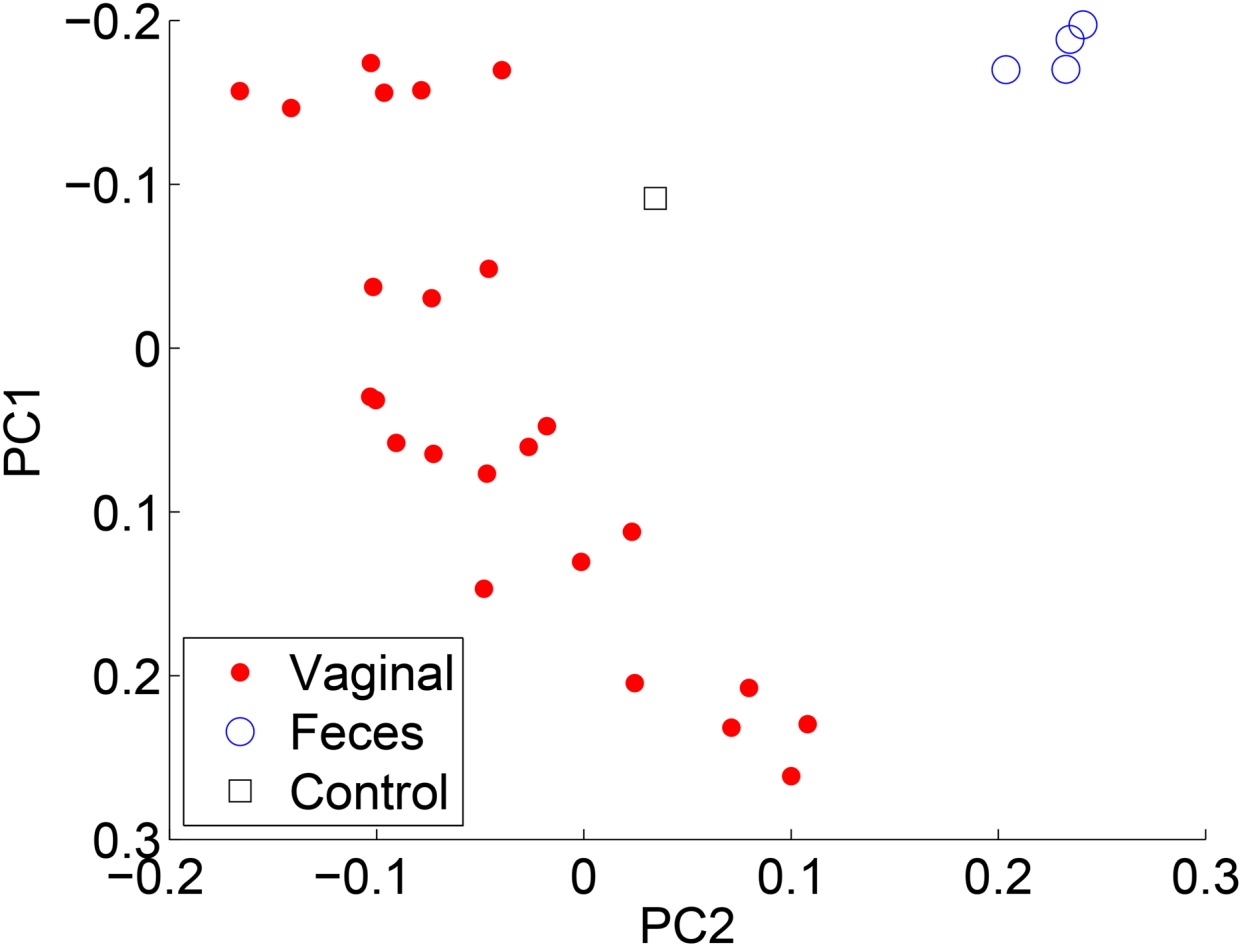
PCoA plots using the Jensen-Shannon divergence for ARK SEK.

To compare the results of each method, we compared PCoA (also known as classical multidimensional scaling) plots by employing the Jensen-Shannon divergence on each of the reconstructions. The points represent individual samples, and the color/shape denote the associated metadata. Each of the methods produced similar PCoA plots. Fig 7 compares the results when using RDP’s NBC and Fig 8 for ARK SEK when the sample body site is labeled. Note the similar clusterings.

**Fig 9:**
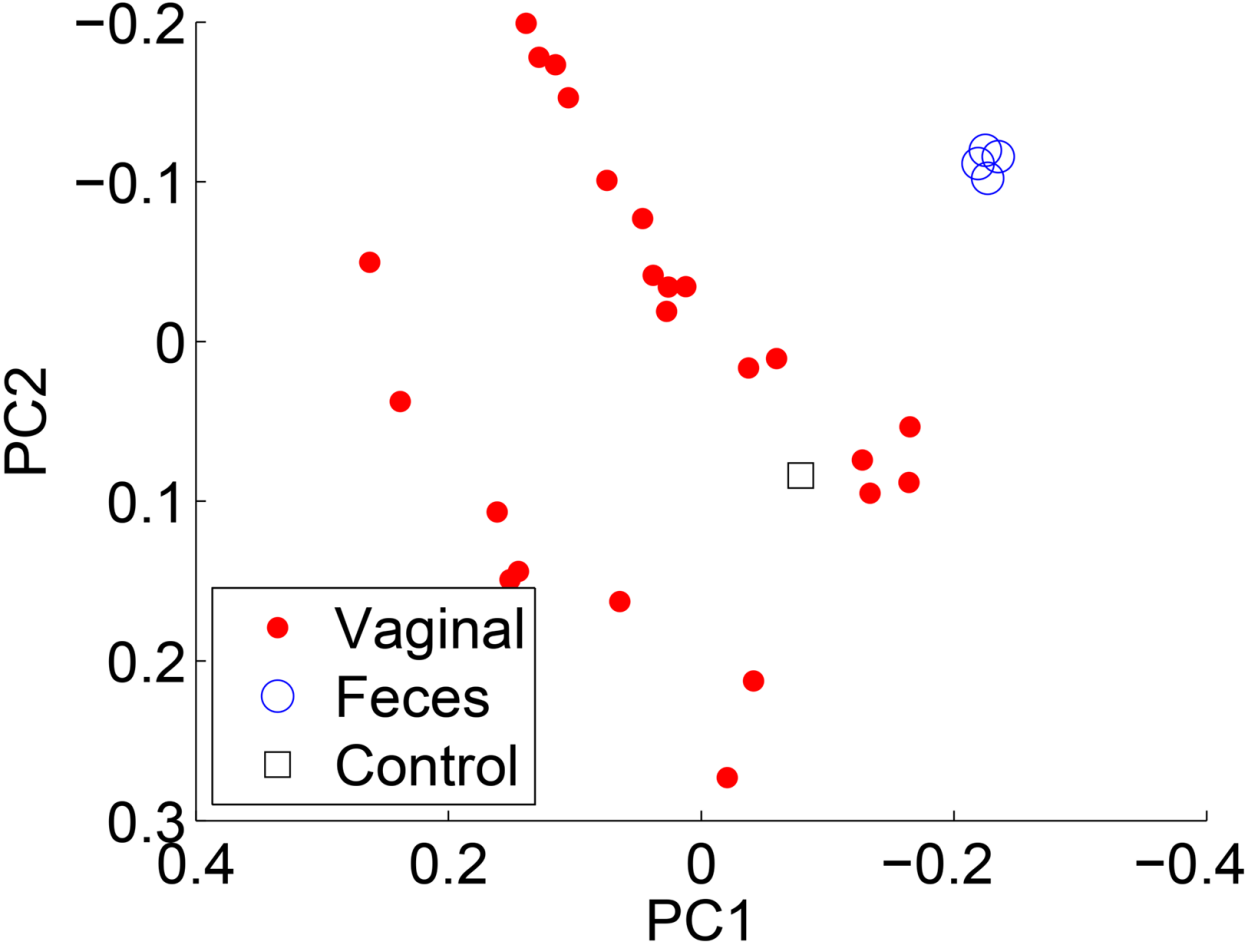
ARK Quikr PCoA plots (using the Jensen-Shannon divergence) on the real biological data. In this case, we have labeling by body site. Note the clustering.

**Fig 10:**
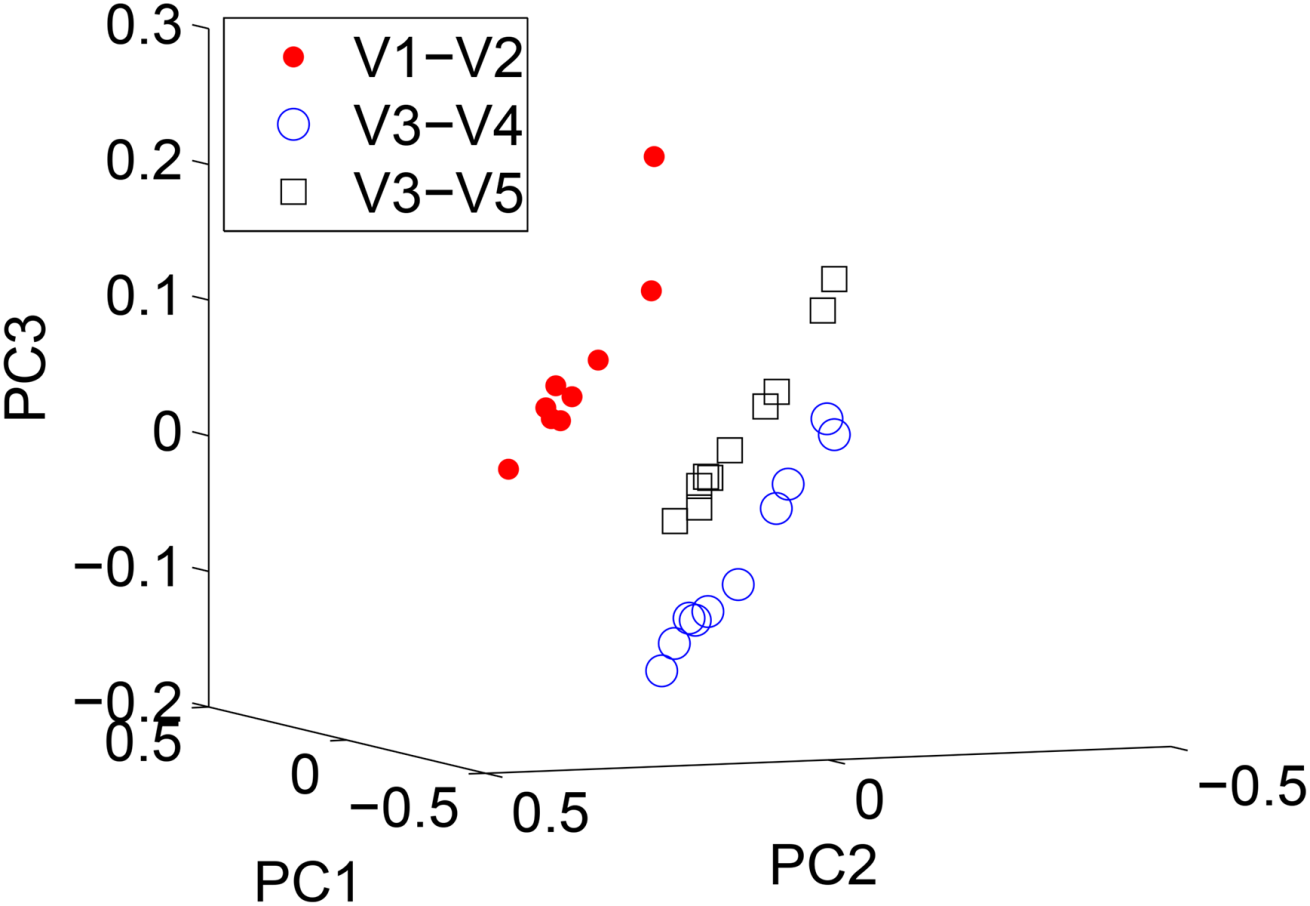
ARK Quikr PCoA plots (using the Jensen-Shannon divergence) on the real biological data. In this case, we have labeling by variable region. Note the clustering.

As shown in Figs 9 and 10, while ARK Quikr gave a somewhat similar PCoA plot with regard to body site (Fig 9), clustering by variable region (Fig 10) was also observed. This is most likely due to the fact that different variable regions have different *k*-mer distributions and different taxa will be preferentially amplified by the varying PCR primers [23]. ARK Quikr can detect this as it analyzes each sample in its entirety, as opposed to the read-by-read nature of RDP’s NBC. This is corroborated by the fact that when using the Jenson-Shannon divergence directly on the 6-mer counts, similar grouping was observed by variable region (results not shown).

## Discussion and Conclusion

The addition of a data processing step based on clustering the read information prior to community composition estimation is akin to the generic divide-and-conquer principle used judiciously in the machine learning field. In terms of information content of the read data, the individual means of the *k*-mer frequencies can collectively provide a better summary than the single mean vector used in the previous approaches, when sufficient heterogeneity is present among the sequences. Our experiments demonstrate this effect by a substantial increase in the accuracy of the resulting estimates. Moreover, the clustering employed by ARK is found to be robust in the sense that it does not lead to lower accuracies, even if a suboptimal number of clusters and clustering strategy were used. We found that the improvement in reconstruction accuracy was obtained at the cost of a moderate increase in execution time for the studied methods.

We note that under the clustering algorithm employed by ARK, no quantitative claims can be made concerning the global optimality of the resulting clusters or on consistent improvement in performance. Also, there is no absolute guarantee that the estimation of *p*(𝒞_*m*_) is bound to improve monotonically with an increase in *Q*. Thus, in an individual experiment, it is possible to encounter occasional degradation in performance. However, our results suggest that a larger number of clusters *Q* will tend to perform reasonably better than a much smaller value of *Q*, provided that the resulting cluster sizes are not too small to yield very noisy estimates of the mean vector.

While this study has focused on 16S rRNA gene sequencing based data, there is no theoretical limitation in applying this technique also to whole-genome shotgun (WGS) metagenomics. Indeed, ARK can readily be combined with existing WGS *k*-mer feature vector metagenomics reconstruction techniques (such as WGSQuikr [24]). Thus, we aim at investigating the versatility of this approach as complementary to other WGS metagenomics analysis methods in the future.

## Supporting Information

S1 File Supplementary Information for “ARK: Aggregation of Reads by K-means for Estimation of Bacterial Community Composition”. This supporting information is available online. This supplementary material is included to address eight major points:
To compare ARK with the best performing bacterial community composition method to date, called BEBaC [8]. BEBaC employs a Bayesian estimation clustering framework along-with a stochastic search and sequence alignment.To investigate the important question of finding the number of regions *Q* in ARK.To independently verify ARK in two different geographic regions ((1) Sweden and Finland, and (2) USA) and also using different datasets.To detail genera-level reconstructions of ARK SEK, ARK Quikr, and RDP’s NBC.To detail the primers used to obtain the data in the main text.To demonstrate the results are qualitatively independent of the error correction method chosen.To detail the effect of changing the *k*-mer size.To investigate the behavior of each method when sister taxa are excluded from the training database.
(PDF)
