# Supplementary material for: ARK: Aggregation of Reads by K-Means for Estimation of Bacterial Community Composition
Source: PLoS One. 2015 Oct 23;10(10):e0140644. doi: 10.1371/journal.pone.0140644 (PMC4619776; doi:10.1371/journal.pone.0140644)
Supplement: S1 File — This supporting information is available online. This supplementary material is included to address eight major points: To compare ARK with the best performing bacterial community composition method to date, called BEBaC [8]. BEBaC employs a Bayesian estimation clustering framework along-with a stochastic search and sequence alignment.To investigate the important question of finding the number of regions Q in ARK.To independently verify ARK in two different geographic regions ((1) Sweden and Finland, and (2) USA) and also using different datasets.To detail genera-level reconstructions of ARK SEK, ARK Quikr, and RDP’s NBC.To detail the primers used to obtain the data in the main text.To demonstrate the results are qualitatively independent of the error correction method chosen.To detail the effect of changing the k-mer size.To investigate the behavior of each method when sister taxa are excluded from the training database. (PDF) [file pone.0140644.s001.pdf]

Supplementary Information for "ARK: Aggregation of Reads by K-means for Estimation of Bacterial Community Composition"

David Koslicki[1], Saikat Chatterjee[2,*], Damon Shahrivar[2], Alan W. Walker[3], Suzanna C. Francis[4], Louise J. Fraser[5], Mikko Vehkaperä[6], Yueheng Lan [7], Jukka Corander [8],

1 Dept of Mathematics, Oregon State University, Corvallis, USA
2 Dept of Communication Theory, KTH Royal Institute of Technology, Stockholm, Sweden
3 Microbiology Group, Rowett Institute of Nutrition and Health, University of Aberdeen, Aberdeen, UK
4 MRC Tropical Epidemiology Group, London School of Hygiene and Tropical Medicine, London, UK
5 Illumina Cambridge Ltd., Chesterford Research Park, Essex, UK
6 Dept of Electronic and Electrical Engineering, University of Sheffield, Sheffield, UK
7 Dept of Physics, Tsinghua University, Beijing, China
8 Dept of Mathematics and Statistics, University of Helsinki, Helsinki, Finland

***To whom correspondence should be addressed. Email: sach@kth.se**

Reason for supplementary data

This supplementary material is included to address eight major points:

1. To compare ARK with the best performing bacterial community composition method to date, called BEBaC [1]. BEBaC employs a Bayesian estimation clustering framework along-with a stochastic search and sequence alignment.
2. To investigate the important question of finding the number of regions Q in ARK.
3. To independently verify ARK in two different geographic regions ((1) Sweden and Finland, and (2) USA) and also using different datasets.
4. To detail genera-level reconstructions of ARK SEK, ARK Quikr, and RDP's NBC.
5. To detail the primers used to obtain the data in the main text.
6. To demonstrate the results are qualitatively independent of the error correction method chosen.
7. To detail the effect of changing the k-mer size.
8. To investigate the behavior of each method when sister taxa are excluded from the training database.

Comparing ARK to BEBaC and finding the number of regions Q

Mock communities data

BEBaC is very accurate, but at the expense of substantial computational cost: a running time of several days in a computing-cluster environment may be required for large read sets. Therefore, to compare ARK with BEBaC, we used a dataset for which the result of BEBaC is readily available. Furthermore, finding Q (the second point above) is also computationally intensive, so the reasonable size of the dataset helped in this regard.

For our experiments on real biological data, we used the mock microbial communities database developed in [2]. The database is called even composition Mock Communities (eMC) for chimeric sequence detection where the bacterial species included are known in advance. Three regions (V1-V3, V3-V5, V6-V9) of the 16S rRNA gene of the eMC were sequenced using 454 sequencing technology in four different sequencing centers. In our experiments we focused on the V3-V5 region datasets, since these have been previously used for the evaluation of the BEBaC method (see Experiment 2 of [1]) and SEK, Quikr and Taxy methods (see Experiment in Section 3.2.2 of [3]).

Test dataset (Reads):

Our basic test dataset used under a variety of different *in silico* experimental conditions is the one used in Experiment 2 of BEBaC [1] and the Experiment in Section 3.2.2 of SEK [3]. The test dataset consists of 91,240 short reads from 21 different species. The length of reads has a range between 450-550 bp and the bacterial community composition is known at the species level by using the following computation performed in [1]. Each individual sequence of the 91,240 read sequences was aligned (local alignment) to all the reference sequences of the reference database and then each read sequence is labeled by the species of the highest scoring reference sequence, followed by computation of the community composition referred to as ground truth.

Training datasets (Reference):

We used a database generated from the eMC database [2]. The database consists of the same $M = 21$ species present among the reads described previously. The details of the reference database can be found in Experiment 2 of BEBaC [1]. The database was also used for training SEK, Quikr and Taxy in the experiment described in Section 3.2.2 of [3]. The database consists of 113 reference sequences for a total of 21 bacterial species, such that each reference sequence represents a distinct 16S rRNA gene. Thus there is a varying number of reference sequences for each of the considered species. Each reference sequence has an approximate length of 1500 bp, and for each species, the corresponding reference sequences are concatenated to a single sequence. The final reference database consists of 21 sequences where each sequence has an approximate length of 5000 bp.

Results

Computational resources and reproducible codes

We used standard Matlab software. For hardware, we used a Dell Latitude E6400 laptop computer with a 3 GHz processor and 8 GB memory. We also used the **cvx** [4] convex optimization toolbox. The reproducible Matlab codes are available at http://www.ee.kth.se/ctsoftware.

Relevant methods

For ARK, we use SEK, Taxy [5] and Quikr as the estimation methods employed to each cluster. The choice of SEK is due to its recent development and good performance. The use of Taxy and Quikr is due to fact that they also use the first order moment of the k-mer frequencies of reads. We use the greedy algorithm, called $\text{OMP}_{\text{sek}}^{+,1}$ to realize SEK. We also use the parameters for $\text{OMP}_{\text{sek}}^{+,1}$ and parameters for k-mers training and testing setups identical to that used in [3] so that fair comparison can be pursued. Finally we compared the performance of ARK-SEK with the ground truth and BEBaC.

Results for Mock Communities data

Using mock communities data, we carried out experiments where the community composition problem was addressed at the species level.

Value of k for k-mers:

Throughout all experiments, we used $k = 4$ for reads and reference. Therefore the k-mer feature vectors have a dimension of 256.

k-mers from test dataset:

In the test dataset, described above, the shortest read is 450 bp long. Therefore, for each read sequence, we used first 450 bp sequence to compute k-mers. Using $k = 4$, the generation of k-mers feature vectors from reads (testset) took 21 minutes of execution time. Note that the k-mers test dataset is same as that used in Section 3.2.2 of [3].

Results:

We first investigated the convergence of ARK and how the performance of ARK behaves with increasing cluster number. We used $\eta = 0.0005$. The performance of ARK using three estimation methods is shown in Figure A. We note that the gross performance trend typically improves with the increase in clusters and then saturates. ARK-SEK saturates at 24 clusters with the VD of 0.0103. The ARK-SEK performance is much better than ARK-Taxy and ARK-Quikr. The scope of improvement of SEK via ARK-SEK is found to be limited. In contrast, Figure B shows that Quikr is found to improve significantly via ARK-Quikr (after convergence).

The convergence time of ARK-SEK was 763 seconds ($\sim$12 minutes). Note that K-means clustering is common for all ARK methods either using SEK, Taxy and Quikr. As ARK-Quikr converged at 17 clusters and took 18 seconds in total, it is clear that the clustering algorithm does not require heavy computational resource. Typically K-means clustering is a simple algorithm and hence can be used for a very large size read dataset without much demand in computational resource.

Finally we compare SEK, ARK-SEK and BEBaC against the ground truth in Figure C. BEBaC results are highly accurate with VD = 0.0038, but come with the requirement of a computation time in the order of more than a day. In comparison, ARK-SEK provides a reasonable performance at the expense of total online computation time of $21 + 12 = 33$ minutes. Therefore, while BEBaC is assumed to incapable of handling a very large dataset of reads, ARK-SEK can be used for this scenario.

Genus-level reconstructions

To provide more detailed insight into how ARK Quikr and ARK SEK perform in comparison to RDP's NBC, we included here genus level reconstructions of a few real data sets (detailed in the main text). We include three samples spanning three variable regions (V1-V2, V3-V5, and V3-V4) and two body sites (vagina and feces). Since each method reconstructs a large number of very low abundance organisms, for simplicity of visualization, we restricted our attention to those genera that appear at an abundance level of $\geq 5\%$ in any of the three methods. Figure D gives a bar chart of each method's genera-level reconstruction on the three data sets. There was a general trend of the ARK SEK reconstruction agreeing more similarly to the RDP NBC results, as was observed in each of the real biological samples described in the main text.

Primer details

For the samples detailed in the main text, 16S rRNA gene amplicons were generated from the DNA extractions using the primer combinations shown in Table 1, where the letters in italics show the region targeting the 16S rRNA gene, non-italicized letters show Illumina adapter, primer pad and linker sequences and "nnnnnnnnnnnn" indicates where unique 12-base Golay barcode sequences were incorporated for each sample.

V1-V2
27f(AATGATACGGCGACCACCGAGATCTACACTATGGTAATTCC*AGMGTTYGATYMTGGCTCAG*)
338r(CAAGCAGAAGACGGCATACGAGATnnnnnnnnnnnnAGTCAGTCAGAA*GCTGCCTCCCGTAGGAGT*)
V3-V4
338f(AATGATACGGCGACCACCGAGATCTACACTATGGTAATTGT*ACTCCTACGGGAGGCAGCAG*)
806r(CAAGCAGAAGACGGCATACGAGATnnnnnnnnnnnnAGTCAGTCAGCC*GGACTACHVGGGTWTCTAAT*)
V3-V5
338f(sequence as shown above)
926r(CAAGCAGAAGACGGCATACGAGATnnnnnnnnnnnnAGTCAGTCAGCC*CCGTCAATTYMTTTRAGT*)

Table 1. Primer combinations

Independence of error correction method

One potential issue is that using a different error-correction method might change the real biological data results contained in the main text. We therefore include in this section PCoA plots for the real biological data using an alternate error correction method, as well as results for using joined paired-end reads.

Read trimming

We used the same computational protocol contained in the main text section on real biological data, but for a different error correction protocol. Namely, we simply trimmed the reads to 150bp. Contained in Figure E are the PCoA plots corresponding to Figures 7, 8, and 9 in the main text. Note the similarity of clustering.

Joining paired-end reads

We used the same computational protocol contained in the main text section on real biological data, but this time we first joined the paired-end sequences (using the ea-utils script fastq-join [6]). Contained in Figure F are the PCoA plots corresponding to Figures 7, 8, and 9 in the main text. Note the similarity of clustering.

Effect of k-mer size

We next consider the question of whether using larger k-mers yields improved performance for Quikr, SEK, ARK Quikr, and ARK SEK. To that end, we utilized the same test datasets (Reads) and Training dataset (Reference) as described in the main manuscript. We then ran each method on all simulated datasets for $k = 4, 5, 6, 7, 8, 9, 10$. For ARK SEK and ARK Quikr, we set the number of clusters to the relatively low value of 10 as a higher number of clusters, combined with larger k-mer sizes, led to prohibitive computational times, particularly for ARK SEK. Figure G demonstrates that each method experiences decreased error as the k-mer size increases, but with diminishing returns (i.e. the decrease in error from $k = 9$ to $k = 10$ is less than from $k = 8$ to $k = 9$). As expected, the execution time of each method increased exponentially as the number of k-mers increases exponentially in k. Figure H gives the execution time of each method. Figure I gives a semi-log plot with the execution time on a logarithmic scale so the relative execution times can be compared.

Given the rapidly increasing execution time combined with the slow decrease in reconstruction error as a function of k-mer size, we recommend using the largest k-mer size as possible that a user's time constraints will allow for.

Excluding sister taxa

We also investigate here how well each method will perform when the testing data has organisms absent from the training database. To this end, we randomly selected one species from each genera in the RDP training set 7 and formed a testing database. Note that in RDP training set 7, some genera consist of a single representative species. The remaining sequences were then used to form a training database. The testing database was then utilized to form 180 simulated datasets using the same simulation parameters described in the main text (under the Test datasets (Reads) section). Each method (RDP, Quikr, SEK, ARK Quikr, and ARK SEK) was retrained using this new, reduced testing database. The parameters utilized for each method were the same as in the main text, and the number of clusters for ARK was set to 75.

Included in figure J is a bar chart of the mean error for each method at the genus level. Compare this with Figure 4 from the main text. As expected, each method experienced an increase in error. The percent increase in mean error at the genus level for RDP, Quikr, SEK, ARK Quikr, and ARK SEK was respectively 3%, 2%, 50%, 12%, and 24%. This is consistent with previous observations [7, Table 1] where it was shown that including novel organisms in the testing data marginally increases the error of Quikr.

However, even given the increase in error of ARK Quikr and ARK SEK, these two methods remain more accurate than RDP's NBC. Indeed, ARK Quikr remains 14% more accurate than RDP, and ARK SEK remains 43% more accurate than RDP.

References

1. Cheng L, Walker AW, Corander J. Bayesian estimation of bacterial community composition from 454 sequencing data. Nucleic Acids Research. 2012;.

2. Haas BJ, Gevers D, Earl AM, Feldgarden M, Ward DV, Giannoukos G, et al. Chimeric 16S rRNA sequence formation and detection in Sanger and 454-pyrosequenced PCR amplicons. Genome Res. 2011;21(3):494–504.

3. Chatterjee S, Koslicki D, Dong S, Innocenti N, Cheng L, Lan Y, et al. SEK: Sparsity exploiting k-mer-based estimation of bacterial community composition. Bioinformatics. 2014;30(17):2423–31.

4. Boyd S, Vandenberghe L. In: Convex optimization. Cambridge University Press; 2004. .

5. Meinicke P, Aßhauer KP, Lingner T. Mixture models for analysis of the taxonomic composition of metagenomes. Bioinformatics. 2011;27(12):1618–1624.

6. Aronesty, E. Comparison of Sequencing Utility Programs. The Open Bioinformatics Journal. 2013; 7:1–8.

7. Koslicki D, Foucart S, Rosen G. Quikr: a method for rapid reconstruction of bacterial communities via compressive sensing. Bioinformatics. 2013;29(17):2096–2102.

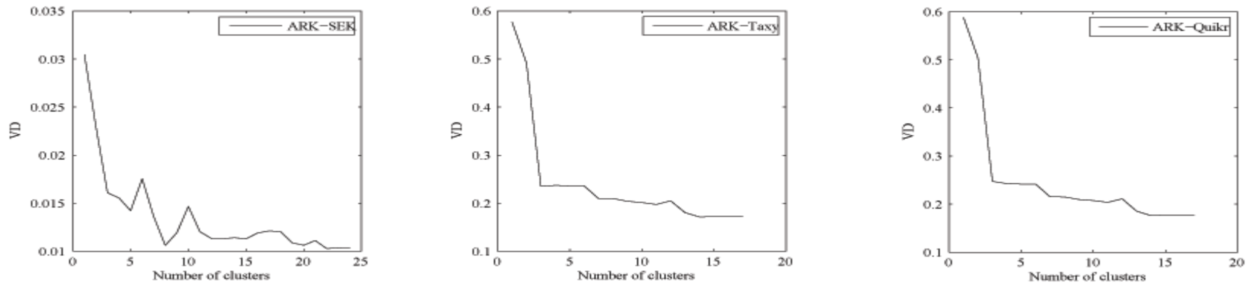

Figure A. For mock communities data: Performance of ARK with respect to number of clusters. The convergence time for ARK-SEK, ARK-Taxy and ARK-Quikr were 763 seconds, 61 seconds and 18 seconds respectively. Note that the ARK-Taxy and ARK-Quikr show significant improvement in performance by the use of clustering.

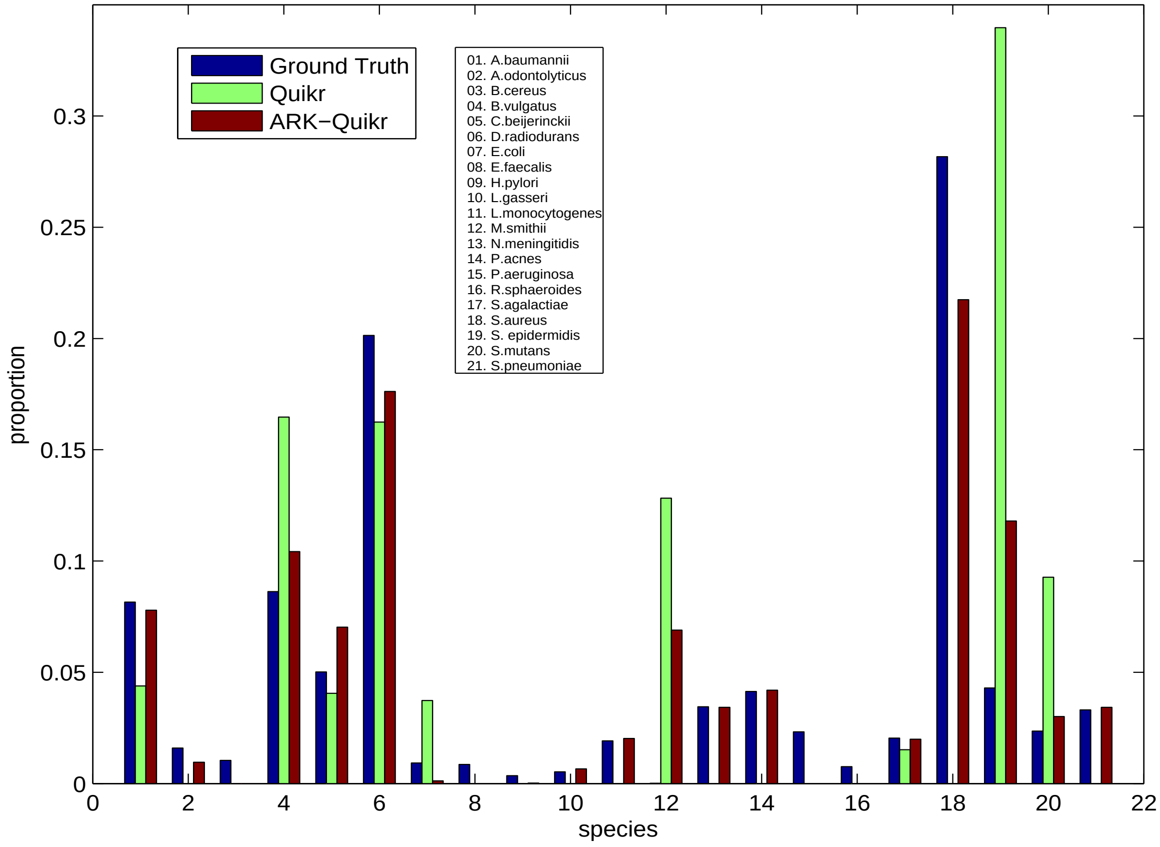

Figure B. Performance improvement of ARK-Quikr vis-a-vis Quikr and ground truth. Quikr and ARK-Quikr produced VD of 0.5882 and 0.1770 respectively.

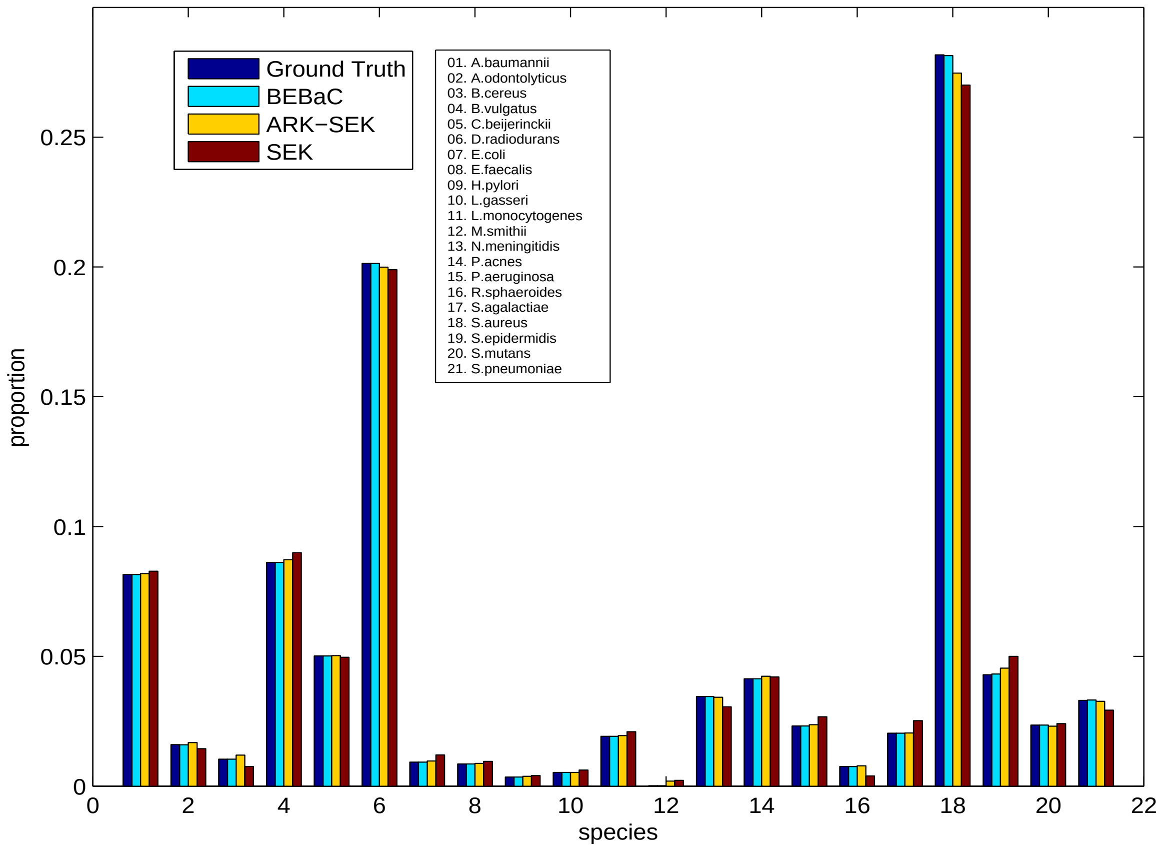

Figure C. Comparison of ARK-SEK, SEK and BEBaC against ground truth. BEBaC, ARK-SEK and SEK provide VD performance of 0.0038, 0.0103 and 0.0305 respectively.

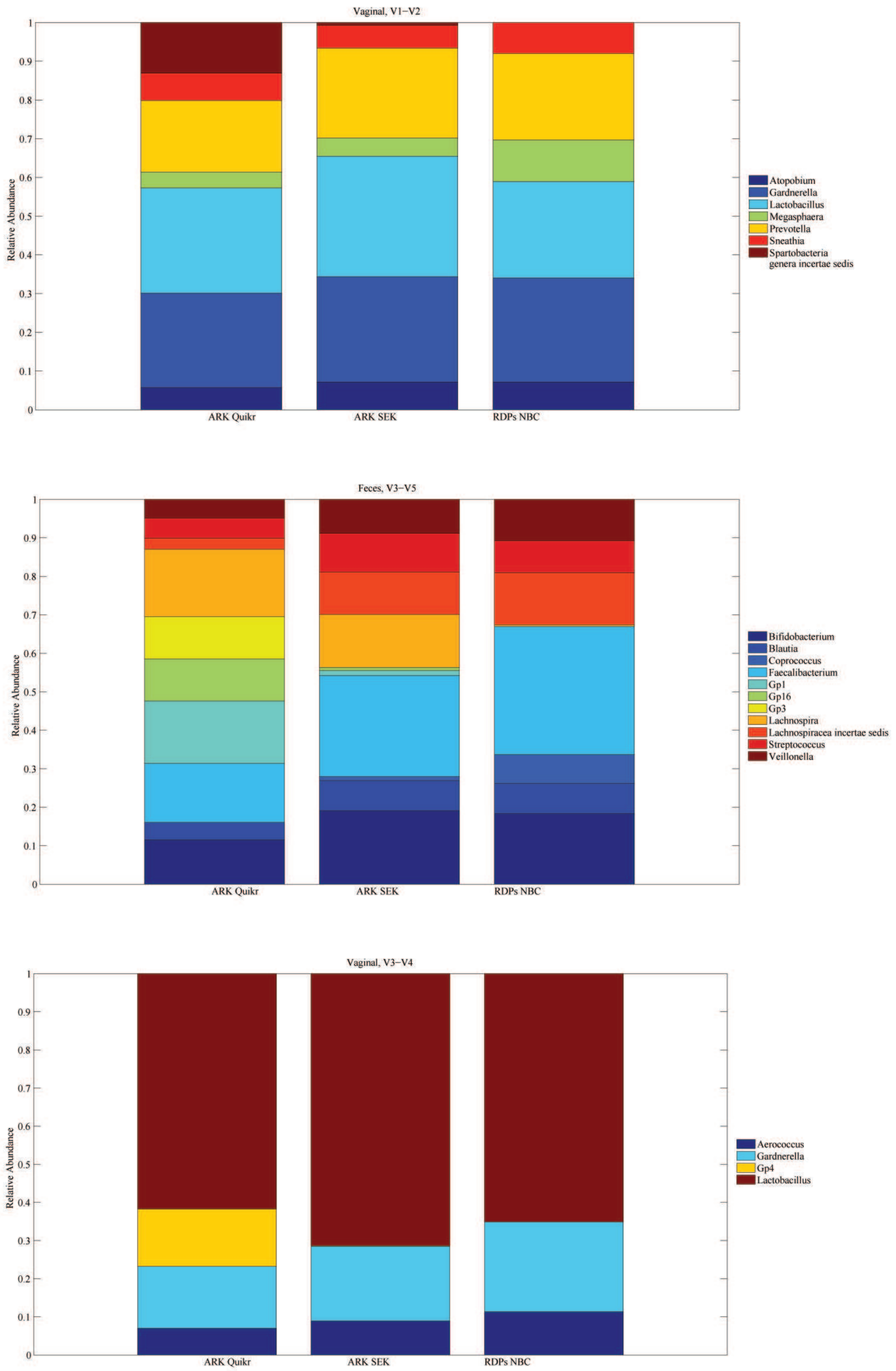

Figure D. Genus level reconstructions of ARK Quikr, ARK SEK, and RDP's NBC on three of the real biological data sets the main text. Genera shown are those that have a proportional abundance of greater than or equal to 5% for at least one of the methods.

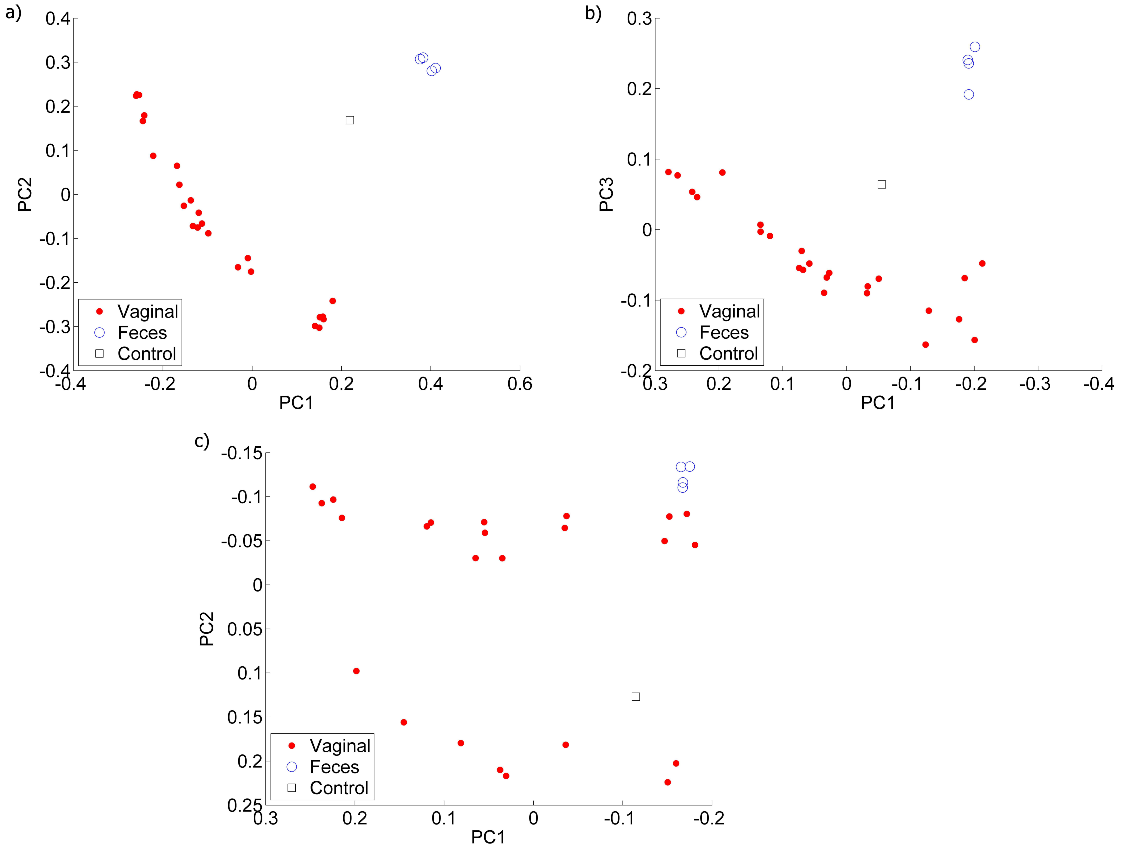

Figure E. PCoA plots using the Jensen-Shannon divergence for the trimmed to 150bp read data with: a) RDP, b) ARK SEK, c) ARK Quikr.

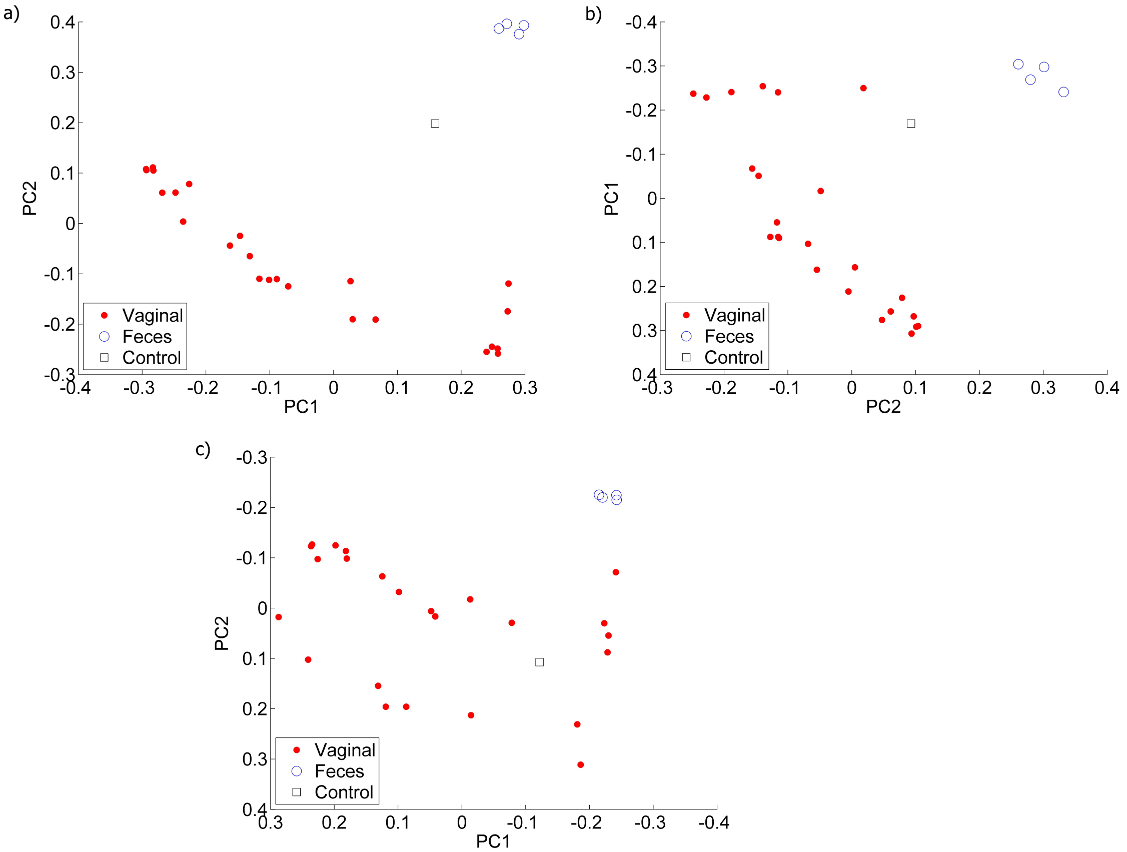

Figure F. PCoA plots using the Jensen-Shannon divergence for the joined paired-end read data with: a) RDP, b) ARK SEK, c) ARK Quikr.

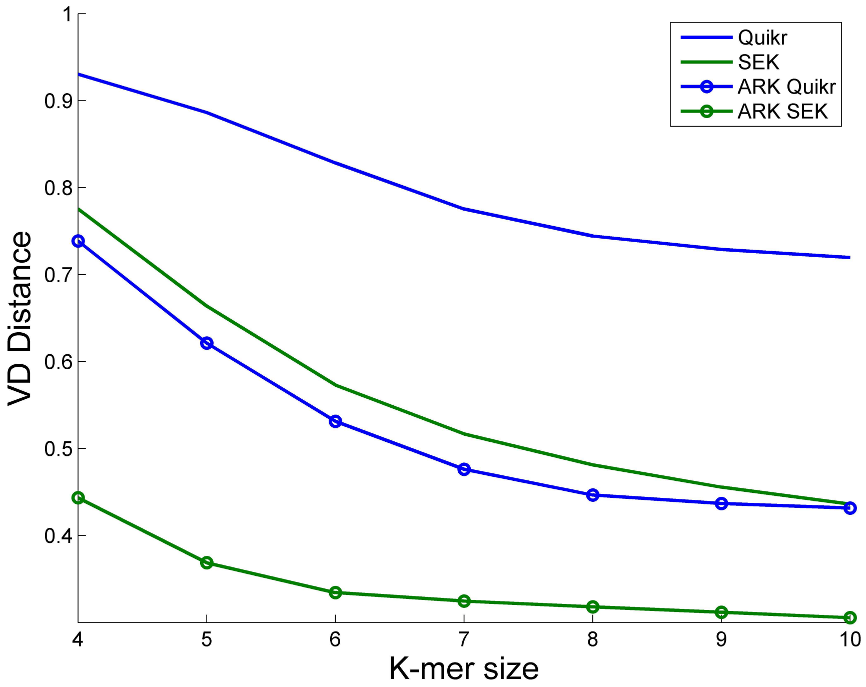

Figure G. Mean genus level reconstruction error for each method as a function of k-mer size.

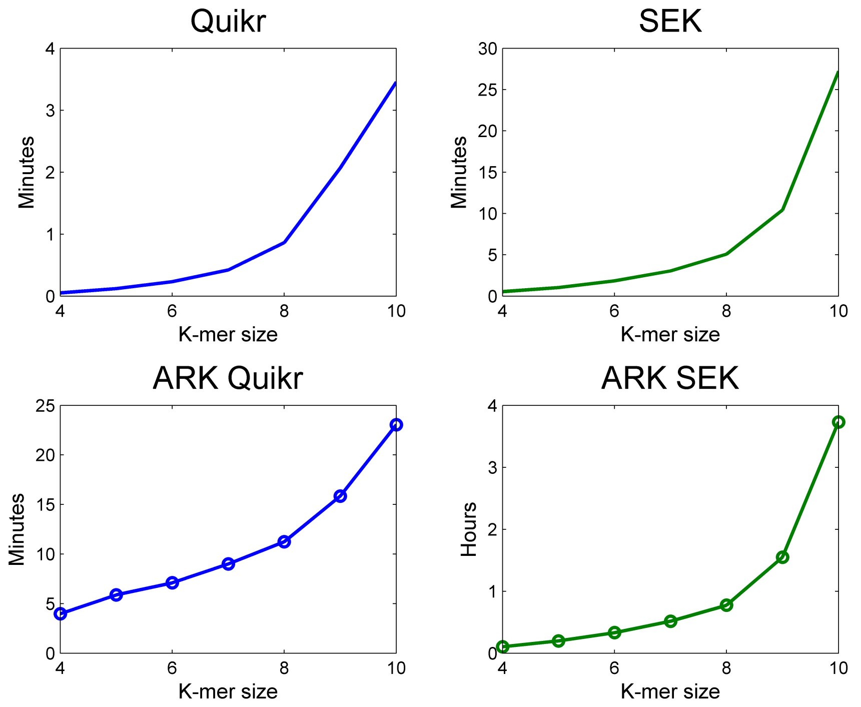

Figure H. Execution time for each method as a function of k-mer size.

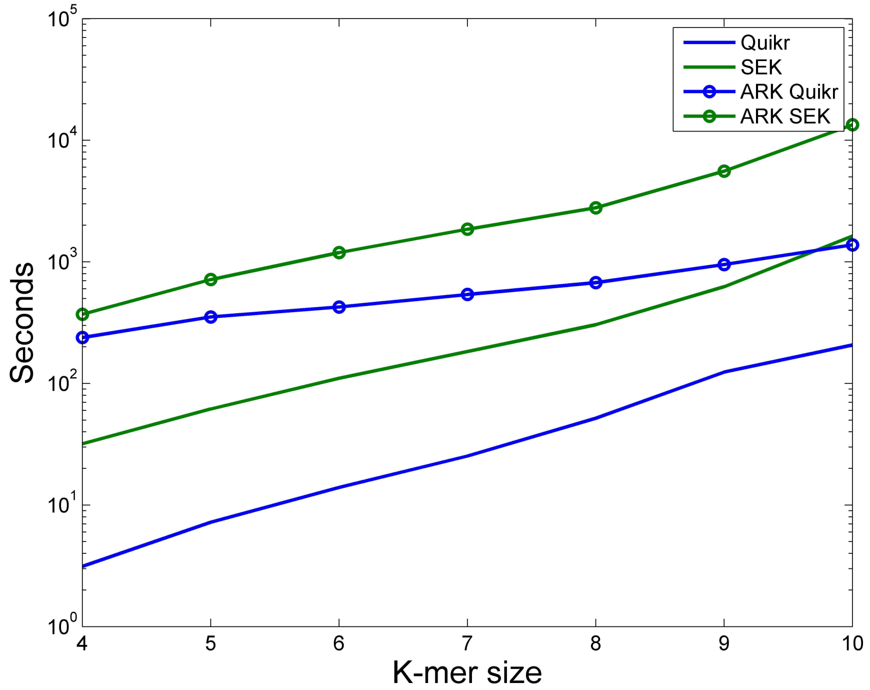

Figure I. Semi-log plot for execution time for each method as a function of k-mer size.

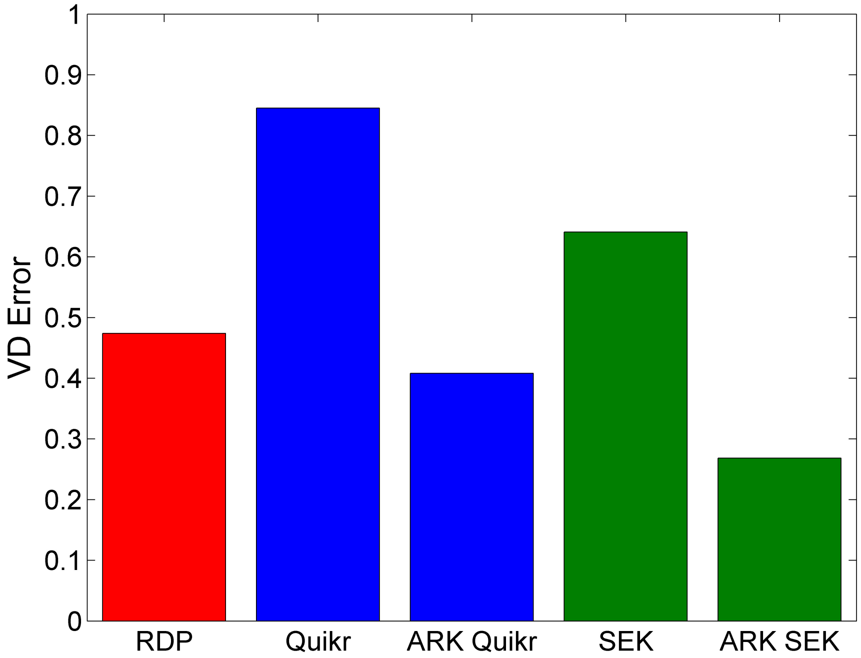

Figure J. Comparison of algorithms when testing data contains organisms absent from the training database. Mean VD error at the genus level.
